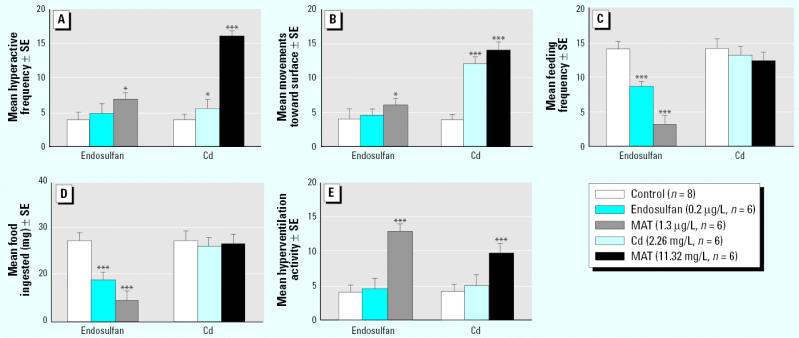# Errata

**Published:** 2005-12

There was an error in Figure 2 of Zeman et al. [ Environ Health Perspect 110:817–822 (2002)]: the *y*-axis should have been labeled “Nitrite” instead of “Nitrate.” The corrected figure appears below.

In Giusi et al. [ Environ Health Perspect 113:1522–1529 (2005)], the colors were incorrect in the key to Figure 1. The corrected figure appears below.

*EHP* regrets the errors.

## Figures and Tables

**Figure:**
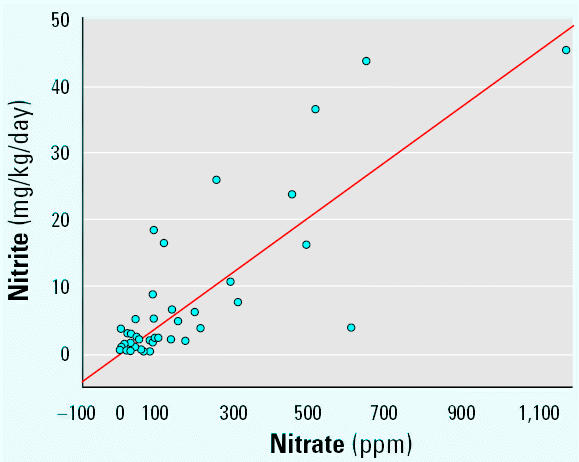


**Figure:**